# Laser Erasing and Rewriting of Flexible Copper Circuits

**DOI:** 10.1007/s40820-021-00714-3

**Published:** 2021-08-31

**Authors:** Xingwen Zhou, Wei Guo, Peng Peng

**Affiliations:** 1 School of Mechanical Engineering and Automation, Beihang University, Beijing, 100191 People’s Republic of China; 2 Department of Mechanical and Mechatronics Engineering, Centre for Advanced Materials Joining, University of Waterloo, Waterloo, ON N2L 3G1 Canada

**Keywords:** Laser writing, Laser erasing, Copper electrodes, Electronic repairing, Flexible electronics

## Abstract

Integrating construction and reconstruction of highly conductive structures into one process is of great interest in developing and manufacturing of electronics, but it is quite challenging because these two involve contradictive additive and subtractive processes. In this work, we report an all-laser mask-less processing technology that integrates manufacturing, modifying, and restoring of highly conductive Cu structures. By traveling a focused laser, the Cu patterns can be fabricated on the flexible substrate, while these as-written patterns can be selectively erased by changing the laser to a defocused state. Subsequently, the fresh patterns with identical conductivity and stability can be rewritten by repeating the writing step. Further, this erasing–rewriting process is also capable of repairing failure patterns, such as oxidation and cracking. Owing to the high controllability of this writing–erasing–rewriting process and its excellent reproducibility for conductive structures, it opens a new avenue for rapid healing and prototyping of electronics.

**Supplementary Information:**

The online version contains supplementary material available at 10.1007/s40820-021-00714-3.

## Highlights

- An up-bottom laser erasing process utilizing electrochemical corrosion has been integrated into the bottom-up writing process.
- The presented erased laser writing technology exhibits excellent reproducibility for sustainable manufacturing of flexible highly conductive Cu structure.
- The suitability of the writing-erasing-rewriting process for repairing failure patterns and reconfiguring circuits has been demonstrated.

**Supplementary Information:**

The online version contains supplementary material available at 10.1007/s40820-021-00714-3.

## Graphical abstract

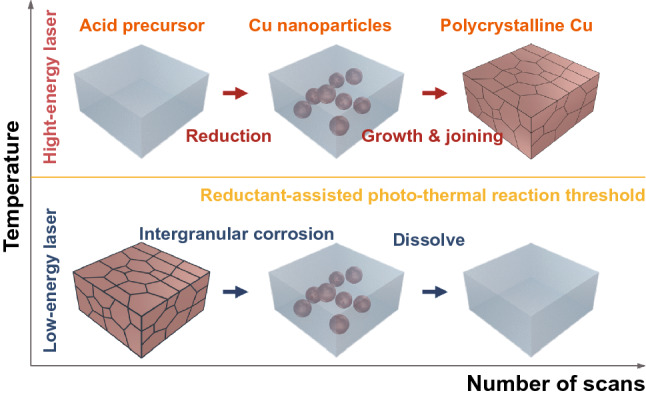

## Introduction

Flexible electronics are indispensable in the fields of healthcare, human–computer interaction, environmental monitoring, artificial intelligence, and energy management [1, 2]. The conductive component is the core part of these electronics as it electrically connects the other electronic components to guarantee the power and signal transmitting in systems [3, 4]. Emerging nanostructured materials open a new avenue for the design and fabrication of these components [4]. The novel efficient manufacturing technologies of conductive structures promote the rapid emerging of high-performance electronics, including various sensors [5–7], energy devices [8–10], and even integrated devices [11–13].

Undoubtedly, prototyping of electronic products is essential for optimizing their performance during the development process [14]. In this process, constant transform of conductive paths is inevitable to adapt to the modified electrical circuits. Reprogramming these circuits in an integrated flexible electronic generally is unrealistic due to a highly integrating of components, resulting in the scrapping of them as defective products. Furthermore, batch-produced electronic failures related to the conductive components are also ubiquitous in the production and service process. Abandonment products in these above processes, together with that have reached the end of economic life [15], result in the generation of electronic waste. Electronic waste management is uneconomical and complicated, and it has arisen a rapidly growing pollution problem worldwide [16, 17]. Therefore, developing an efficient technology suitable for flexible electronics restoring is in high demand.

Reversible laser processing of nanostructured materials is expected to be an alternative solution to this proposition because it integrates the construction and reconstruction processes, which has recently attracted considerable interest in information storage, dynamic coloration, and reprogrammable functional resistance manufacturing [18–22]. The basic principle of these techniques is based on the interconversion of nanostructured metals and oxides, which can be controlled by varying the process parameters such as atmosphere, laser parameters, laser type, and precursor composition [19–22]. However, manufacturing highly conductive components with these methods is rarely reported, probably because these reversible structures' conductivity is not satisfactory. Although with the assistance of reducing agents, a photothermal chemical reduction can selectively reduce oxidized conductive structures to restore their electrical conductivity; this process is only suitable for the nanomaterials with high specific surface area (such as nanowires) [23, 24]. This suggests that the process is based on the material prepared by complex pre-synthesis technologies, and the conductivity of the obtained structures still can be further improved because of their porosity.

Here, we introduce an all-laser processing technology that utilizes the interaction of the laser and a low-cost liquid precursor to write, erase, and rewrite the highly conductive Cu patterns on the flexible substrate. The structural evolution during processing and the underlying mechanism of laser erasing is investigated. The erasing process can selectively remove the partial or complete written structure to restore the substrate to a near-initial state, which is also capable of repairing the oxidized or cracked failure structures. The similar electrical performance and stability between the written and rewritten patterns can be achieved. Because the construction and removal of Cu patterns can be dynamically switched via adjusting the laser parameters during processing; the application of this technology in the prototyping of electronics is also demonstrated.

## Experimental

### Precursor Preparation and Laser Processing

In a typical procedure for preparing the Cu ionic precursor, copper nitrate trihydrate (Cu(NO_3_)_2_·3H_2_O) was dissolved in N-Methyl pyrrolidone (NMP) solution (3 mol L^−1^) and then mixed with ethylene glycol (EG) at a volume ratio of 10:1. Before laser processing, the 200 μm-thick polyimide (PI) and 1 mm-thick glass substrates were washed and hydrophilized with O_2_-plasma. Subsequently, 600 μL of the precursor was covered on the desired region of substrate (25 × 50 mm, Fig. S1). A continuous diode laser system (BWT Beijing Ltd) of a wavelength of 808 nm was used as a laser source for processing. All the laser processing was performed under ambient conditions with a constant scanning velocity of 10 mm s^−1^. The laser power used for writing and erasing was set to 4.7 and 2.7 W, respectively. An objective lens (4 ×) was used to focus the laser beam to around 650 µm (measured using standard photographic paper) for writing. Defocus of laser in erasing was achieved by adjusting the distance between the objective lens and the substrate.

### Material Characterization

UV–vis spectra of the liquid precursors were determined using a UV–vis spectrometer (Cary 5000, USA Varian). Viscosity of the precursor was characterized using a rheometer (MCR92, Anton Paar) at room temperature (~ 25 °C). Differential scanning calorimetry and thermal gravimetric analysis (DSC–TGA) of the precursor were carried out with a SDT Q600 thermal analyzer with a heating rate of 10 °C min^−1^ under air atmosphere. The microstructure and morphology of the patterns were characterized by optical microscope (OM, Zeiss Scope.A1), field emission scanning electron microscopy (FESEM, Merlin Compact), and transmission electron microscopy (TEM, JEOL 2100). Crystal structures were measured by an X-ray diffraction (XRD, Cu Kα radiation, D8-Advance, Bruker, USA). X-ray photoelectron spectroscopy (XPS, K-Alpha, ULVAC-PHI PHI-5000 VPIII, Japan) in conjunction with Ar^+^-ion sputtering was used to characterize the approximate element composition of the surface as a function of depth. All the XPS spectra were corrected using the C 1 s peaks (284.8 eV) as reference. Electrochemical test was performed on an electrochemical workstation (CHI660E, CH Instruments, Shanghai) in a HNO_3_ solution (pH = 2.5) using the written patterns as working electrode. The commercial platinum foil (2.5 × 4 mm) and Ag/AgCl (saturated KCl) electrode were used as counter electrode and reference electrode, respectively. The pH values of the solutions were measured by pH meter (PHB-4, INESA, Shanghai) with E-201F electrode.

### Electrical Performance, Mechanical Stability, and Thermal Stability Tests

All the electrical performances were carried by a Keithley 2400 source-meter. The bending performance of the fabricated pattern was performed using a two-axis platform with a controllable velocity. The experimental setup was established by moving one end, while the other end was fixed. The in situ resistance change of pattern during bending was monitored by connecting additional wrapping wires at the ends of patterns. The adhesion test of patterns was performed based on a tape peeling test; 3 M tape was pressed onto the top of the patterns and then manually torn off. The thermal stability was conducted in a conventional oven with a controllable temperature environment.

## Results and Discussion

**Fig. 1:**
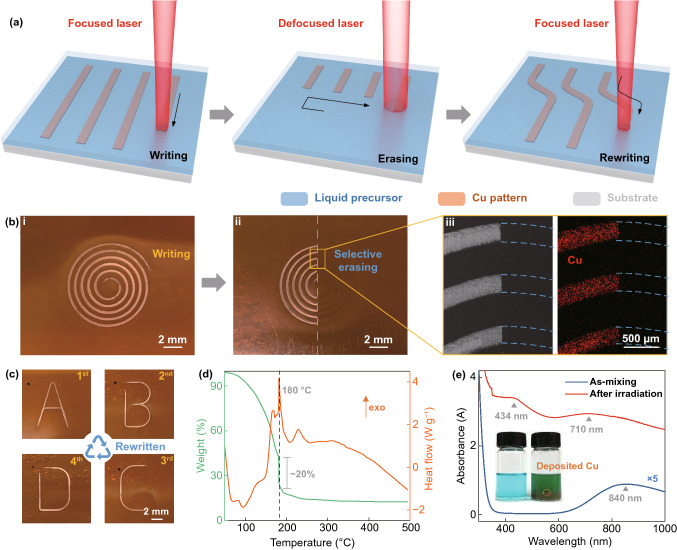
Demonstration of erasable laser writing process and the precursor characterization. **a** Schematic of the all-laser writing–erasing–rewriting process. **b** Characterization of the pattern during writing–erasing process. Digital photos of the (i) as-written pattern (ii) after selective erasing, and (iii) SEM and EDS images. **c** Letters successively written and erased at a same region on the substrate. The black dot is a deliberate mark for this region. **d** DSC and TGA curves of the precursor. **e** UV–vis spectra of the precursor (diluted to 1/200 using EG) before and after laser irradiation. Inset photos show the solution change after irradiation, whereof the dotted circle marks the deposited Cu on the glass bottle

Figure 1a illustrates a schematic of the proposed laser writing–erasing–rewriting technology, in which all the processes are performed by irradiating a traveled laser on the substrate covered with a liquid Cu salt precursor. The Cu pattern is fabricated by scanning a focused laser at the desired area, while the written pattern can be selectively erased by irradiating a defocused laser. Subsequently, the writing process can be executed again with the focused laser to fabricate a new Cu pattern. Figure 1b demonstrates this writing and erasing processes on a commercial PI substrate. An Archimedean spiral Cu pattern is first written by scanning the focused laser (Fig. 1b-i), and part of this pattern is then erased by scanning with a defocused laser (Fig. 1b-ii). Figure 1b-iii shows SEM and EDS characterizations of the interface between written and erased regions. The pattern and corresponding Cu element are invisible in the erased region, while it still exists intact in the written region, confirming the excellent selectivity of the erasing process. There is no doubt that the written pattern can be fully erased via controlling the defocused laser trajectory. By replacing the precursor after each writing–erasing cycle, the rewriting process can be multiple performed, as demonstrated by the sequential patterning of "A-D" letters on the same area of the PI substrate (Fig. 1c). Only the current letter can be observed at this region, indicating the previous pattern has been completely removed after each erasing cycle. The similar morphologies and lusters of these patterns confirm the good reproducibility of the rewriting process, also suggesting negligible damage to the substrate of these multiple erasing–rewriting cycles.

The used precursor is a modified solution from our previous study [9], which is a low-cost solution consisting of Cu(NO_3_)_2_·3H_2_O and EG dissolved in NMP. The viscosity of the precursor in the range of 10–100 s^−1^ is constant at 225.2 mPa s, indicating it is a Newtonian liquid with ideally viscous. Figure 1d plots the DSC–TGA curves of the precursor. As seen, the endothermic peaks at around 202 and 87 °C are, respectively, attributed to the evaporation of NMP and water from Cu(NO_3_)_2_·3H_2_O [25, 26], while the exothermic peak at around 212 °C is related to the decomposition of Cu(NO_3_)_2_ [26]. Importantly, the peaks at around 180 °C (with a range from 140 to 200 °C) is identified in the DSC curve, which agrees to the decomposition temperature of EG [27, 28]. Correspondingly, an obviously mass loss of about 20% is observed in the TGA curve, suggesting the formation of Cu structures [24, 29]. It can be considered that thermal decomposition of the reducing agent accounts for the Cu^2+^ reduction to assist the laser writing process. The polyol reduction mechanism can be summarized as [24, 30]:

$$ 2{\text{HO}}({\text{CH}}_{2} )_{2} \;\xrightarrow{{\left( {{\text{heat}}} \right)\, - \,2{\text{H}}_{2} {\text{O}}}}2{\text{C}}_{2} {\text{H}}_{4} {\text{O}}\,\;\xrightarrow{{{\text{Cu}}({\text{II}})}}{\text{C}}_{4} {\text{H}}_{6} {\text{O}}_{2} \, + \;{\text{H}}_{2} {\text{O}}\; + \;{\text{Cu}} $$

As the precursor can absorb laser in near-infrared wavelength range around 840 nm (Fig. 1e), irradiating with a laser in the corresponding wavelength range (808 nm laser used in this work) can directly heat the precursor. This also allows a wider selection of substrates for processing (for example the glass substrate, see Fig. S2). Infrared imaging indicates the region around laser spot can be higher than 180 ℃ during irradiating, while it is only around 120 ℃ in the solution bulk (for detailed information, see Fig. S3). After irradiating, a thin Cu layer will be deposited on the inner wall of the glass bottle while the color of solution turns from blue to dark green (as marked in inset of Fig. 1e). The UV–vis spectrum indicates the absorbance of precursor obviously increases after laser irradiating, and two peaks at 434 and 710 nm can be observed. The former one closes to the intrinsic bandgap absorption of Cu_2_O [31, 32], while the latter one may be assignable to the transformation of Cu^2+^ complexes [33, 34]. This confirms that the amount of Cu formed in the solution bulk is almost negligible due to the insufficient temperature (also can be verified by the XRD analysis, see Fig. S4). These results indicate the laser irradiation will only cause a local temperature rise if the processing is performed on a target substrate, which is beneficial to avoid global thermal damage to the flexible substrate (the effects of substrate on the thermal accumulation have been discussed in supporting information, see Fig. S5).

**Fig. 2:**
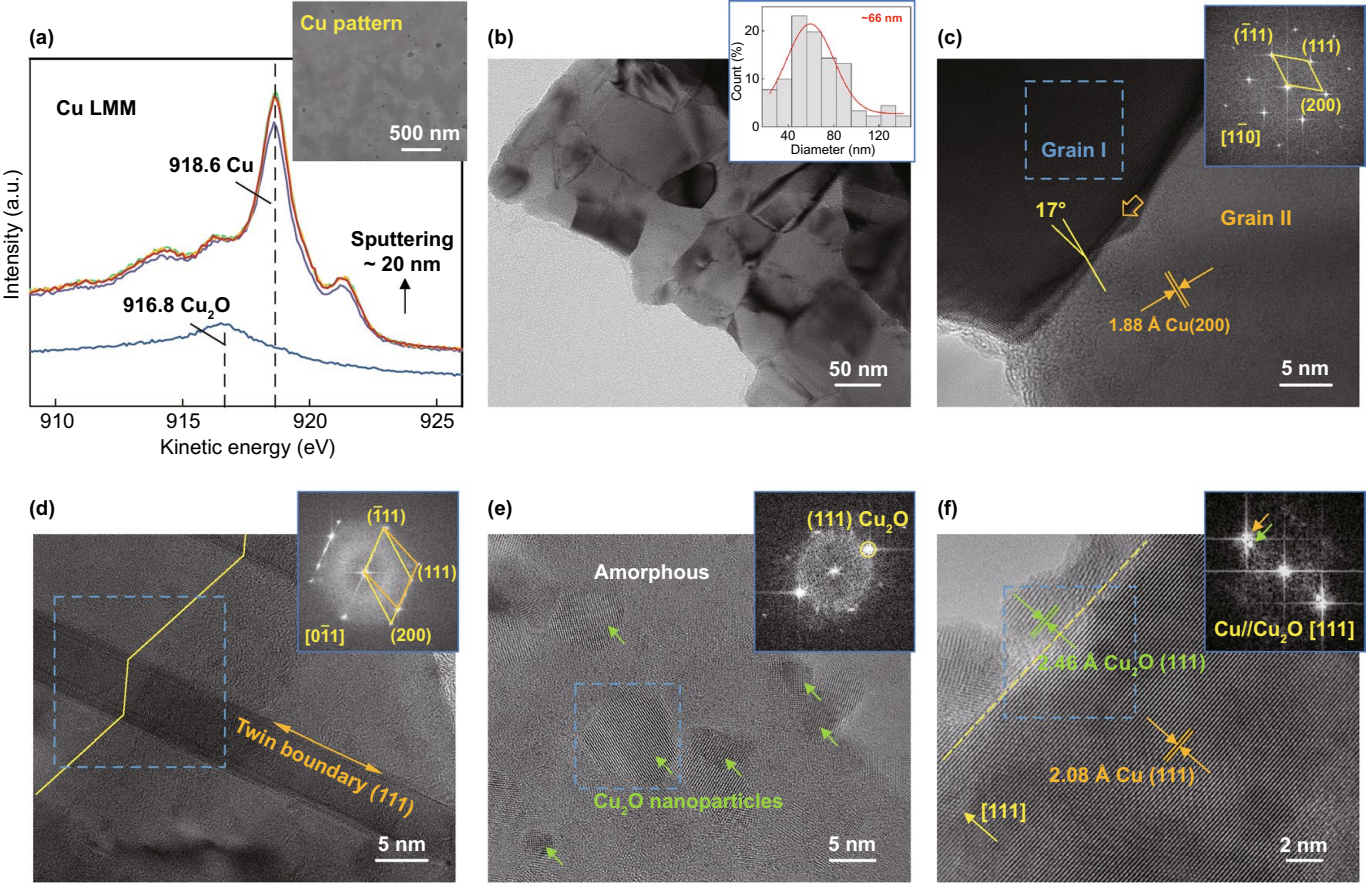
Characterization of the typical written pattern. **a** Cu LMM spectra of the pattern obtained at 10 *N*_w_ as a function of the sputter depth. Inset SEM image shows the morphology of the typical pattern. TEM images of the **b**–**d** typical dense Cu and **e**–**f** Cu_2_O structures in the pattern. Inset image in b is the statistics of the particle size. Insets in **c**–**f** are the FFT images corresponding to the regions marked by the blue dotted boxes

According to our previous study [9], the resistivity of the written pattern can reach an excellent value only ~ 2.5 times that of bulk Cu (around 4 × 10^–8^ Ω m), which is superior to the previously reported flexible structure obtained by one-step laser writing. Here, the writing parameters are further optimized to improve the writing efficiency (for detail parametric study, see Section S3 in Supporting Information), which can fabricate the pattern with a similar order of magnitude as bulk Cu after five laser scans (labeled as *N*_w_). Figure 2a plots the Cu LMM spectra of the typical pattern at different sputter depths. A peak at 916.8 eV [35] is identified on the surface, indicating the chemical state of Cu on the surface is mainly of Cu_2_O. Increasing the sputter depth larger than ~ 5 nm, only the Cu peak at around 918.6 eV [35] can be observed. The peak position is constant with further increasing the sputter depth, confirming the Cu_2_O only present within ~ 5 nm near the pattern surface (for atomic concentration at different sputter depth, Fig. S8). Obviously, high Cu content and dense structure of pattern (inset in Fig. 2a) account for its excellent conductivity.

The crystallographic characteristics of pattern are further analyzed by TEM observation. The obtained pattern mainly consists of a dense sintered polycrystalline structure with an average grain size of 66 nm (Fig. 2b). High-resolution TEM observation indicates these single crystalline grains are with a clear lattice spacing of 0.208 nm corresponding to the (111) plane of cubic Cu, which also can be verified by the fast Fourier transform (FFT) analysis (Fig. 2c). These grains are mainly interconnected via random boundaries, such as a high angle boundary with a misorientation angle of about 17°. The Ʃ3 boundaries (growth twins parallel to each other in {111} planes with the semi-coherent interface) are also identified (Fig. 2d), which are common in the epitaxially joined cubic nanostructures due to their low stacking-fault energy [36, 37]. Pulsed thermal cycle associating with multiple laser scans limits the grain size in nanometers because instantaneous high-energy input is beneficial to promote the formation of nucleation sites, while rapid cooling promptly interrupts grain growth and facilitates the formation of growth twins [38]. Consistent with the Cu LMM analysis, a few Cu_2_O can be identified in TEM observation (almost all identified lattice spacing is (111) plane, 0.246 nm), which, respectively, is introduced by insufficient reduction in Cu^2+^ species and re-oxidation of Cu structures. The former is the independent nascent Cu_2_O nanoparticles (about a few nanometers in diameter) surrounded by a large amount of residual amorphous organics (Fig. 2e), while the latter is grown on the Cu matrix surface with a similar orientation (about 4 nm-thick, Fig. 2f).

**Fig. 3:**
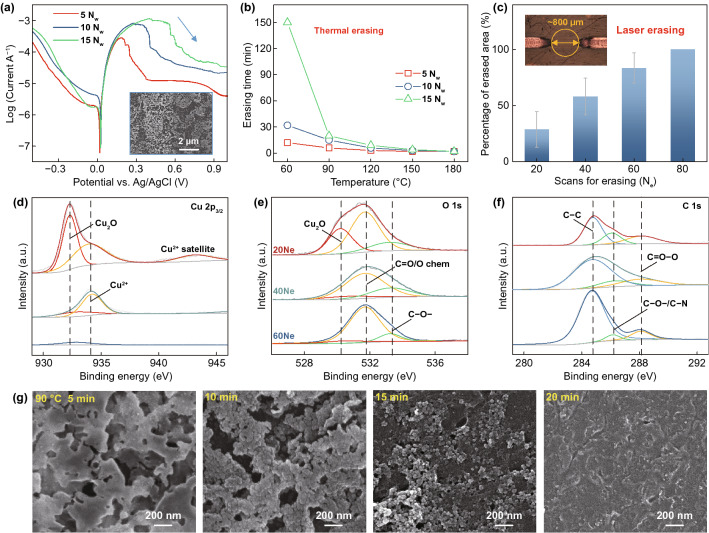
Erasing mechanism and structural change. **a** Tafel polarization curves of the patterns obtained at different *N*_w_ in HNO_3_ solution (pH = 2.5). Inset SEM image shows the morphology of the typical pattern after electrochemical test. **b** Erasing time of the patterns obtained at different *N*_w_ during thermal erasing with various temperatures. **c** Erased area percentage of the pattern obtained at 10 *N*_w_ as a function of Ne. Inset shows the morphology of the pattern obtained at 10 *N*_w_ with a full erasing area of about 0.25 mm^2^ after 80 *N*_e_ **d** Compositions of the pattern as a function of *N*_e_. **e** Surface morphologies change of the pattern during thermal erasing

The pH value of our precursor is around 2.5 due to the presence of H^+^ after Cu(NO_3_)_2_ hydrolysis. The erasing process is enabled through electrochemical corrosion of the written Cu pattern in this natural acid medium. To confirm this mechanism, Fig. 3a plots the polarization curves of the written patterns in HNO_3_ solution with a pH of 2.5. The Cu pattern is corroded to Cu^2+^ in HNO_3_ solution without the formation of passivation CuO layer, thereby no steep slope appears in the anodic range [39]. The patterns with different *N*_w_ have a similar corrosion potential at 0.025 V (vs. Ag/AgCl), suggesting their similar corrosion resistance. Interestingly, in the anode range the current first increases and then decreases as elevating the potential. This abnormal decrease in current is because the dissolution of Cu (inset image in Fig. 3a) results in the disconnection of working electrode. The potential value of the inflection points increases as the *N*_w_ grows, indicating the dissolution is positively correlated with the volume of written Cu pattern.

Although the external voltage load can promote the dissolution of the pattern, it is difficult to completely erase it because the increased resistance will significantly slow down the corrosion. It is found that heating in the acid precursor can accelerate the corrosion process and completely erase the written pattern. Figure 3b plots the erasing time as a function of heating temperature, clearly showing a higher temperature can shorten the erasing time of pattern. At a similar temperature, the thermal erasing time increases as the *N*_w_ grows because of the increased Cu pattern volume. Obviously, bulk heating of patterns in solution is a non-selective process, which can be used in erasing in large area.

In this work, we use laser irradiation to selectively erase the undesired area of the pattern. The width of the erased region is limited to about 800 μm when irradiating at a defocus distance of 9 mm (see inset of Fig. 3c). Figure 3c plots the percentage of erased area relative to the initial patterned area at this region. The removed area increases as the number of erasing scans (*N*_e_) increases, whereof the pattern can be fully erased after 80 *N*_e_. The area of the erased region is related to the laser spot diameter, which can be decreased by reducing the defocus distance. Notably, an excessive decrease in the spot diameter with a constant laser power is inadvisable, because the increased energy input will reperform the writing process. The reintroduced Cu structure is easily oxidized into CuO because the liquid film rupture caused by the large-spot laser irradiation [40] will expose the structure to the atmosphere at high temperatures (detailed results and analysis of parametric study are in Section S4 of Supporting Information**)**. This CuO also can act as the passive layer to slow down the corrosion [41], causing the erasing process is no longer applicable.

Figure 3d lists the XPS spectra of the Cu pattern with different *N*_e_ to track its composition change during erasing. The intensity of Cu 2p_3/2_ peaks decreases as the *N*_e_ increases, confirming the dissolution of pattern. The dominant peak at 932.6 eV is assigned to the Cu_2_O because a corresponding peak at around 530.7 eV can be observed in the O 1 s spectrum [42]. Pure Cu is hard to be observed during the corrosion process because the dissolution of Cu first forms the absorbed Cu^1+^ species on the surface [43]. Laser heating of the precursor during erasing may also introduce few newly Cu_2_O_._ The subpeak at 934.7 eV may be related to the subsequent formation of soluble Cu^2+^ species [44]. Its corresponding peak located at around 531.8 eV in the O 1 s spectrum, which coincides with the C=O peak [45, 46]. These results agree well with the dissolution steps of Cu in nitric acid, which can be described as ${\text{Cu}} - {\text{e}}^{ - } \to {\text{Cu}}\left( {\text{I}} \right)_{{{\text{ads}}}}$ and ${\text{Cu}}\left( {\text{I}} \right)_{{{\text{ads}}}} - {\text{e}}^{ - } \to {\text{Cu}}({\text{II}})$ [43, 47]. The C–O peak located at 533 eV is also identified [48]. Both the C=O and C–O bonds are attributed to the presence of residual organics [49, 50], which are chemically adsorbed on the pattern surface. In the C 1 s spectrum, the peaks at 284.8, 286, and 288.2 eV are, respectively, assigned to the C–C, C–O/C–N, and C=O bonds [51, 52]. The atomic concentration of C–O/C–N and C=O bonds decreases from 44.62 to 29.03% as the *N*_e_ increases (calculated based on their peak areas relative to that of the C–C bond), suggesting these adsorptions decrease as the pattern dissolves. Finally, the composition at the erased region is close to the fresh PI substrate (with only 0.15% atomic concentration of Cu remains, see Fig. S13).

Figure 3e displays the typical morphologies of Cu pattern during the thermal erasing process to understand its microstructure change. After heating at 90 °C for 5 min, the dense pattern turns into a porous structure due to its partial dissolution. Nanoparticles with sintered necks are visible as the heating time increases to 10 min. The mean diameter of these nanoparticles is 60 nm, which closes to the grain size in TEM observation as previously shown in Fig. 2e. This indicates the intergranular corrosion occurs first during the erasing process because of a large number of defects at the grain boundary (the arrow marked in Fig. 2e). With increasing the heating time to 15 min, the separated nanoparticles are exposed and their number decreases due to the continuous dissolution of pattern. The Cu nanoparticles will be invisible after heating for 20 min, leaving only the PI substrate present. The erased region of the substrate is slightly rougher than the fresh region (Fig. S14) because the thermal conducted from the precursor during the writing process can cause a slight ablation of the PI surface. The microstructural evolution of the pattern during the laser erasing process shows no difference with thermal erasing, excepting that the characteristics of intergranular corrosion are harder to be identified (Fig. S10). This may be because the temperature when erasing with the selected laser parameters is greater than 90 °C, which introduces more adsorbed organics.

**Fig. 4:**
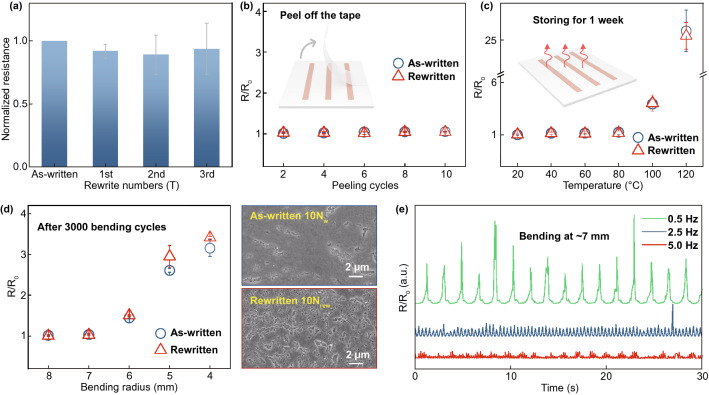
Electrical performance and stability of the first-written and rewritten structures. **a** Normalized resistance changes of the structures as a function of rewritten numbers. Relative resistance changes of the structures after **b** adhesion testing, **c** thermal stability testing, and **d** bending testing. Insets in d are the surface morphologies of the as-written and rewritten patterns. Both the *N*_w_ and *N*_rew_ are 10. **e** In situ relative resistance changes of the typical pattern during the bending cycles. Different in *R*/*R*_0_ value is because the change rate exceeds the 0.04 s sampling interval

Figure 4a compares the normalized resistance of the first-written and rewritten Cu patterns. After 10 scans of rewriting (*N*_rew_), the average normalized resistance of the pattern slightly decreases and will remain around 90% compared to the initial one. This may be attributed to the certain roughness at the surface after erasing can provides more sites for Cu nucleation, thus slightly increasing the volume of Cu pattern [53]. The rewritten pattern can reach similar stability toward the as-written pattern. Figure 4b compares the relative resistance change of these patterns during adhesion testing with 3 M adhesive tape. The resistances of patterns are almost constant after 10 peeling cycles, indicating the excellent adhesion between pattern and substrate. Notably, this also suggests the adhesive tape can be used in thermal erasing to selectively protect and erase the patterns. By attaching 3 M tape to the area to be retained, only the pattern that directly touched the precursor can be erased during the thermal erasing process (Fig. S15).

Figure 4c plots the relative resistance changes of these patterns after storing at different temperatures for 1 week. The relative resistance change of the rewritten pattern is equivalent to that of the as first-written pattern. The resistance of these patterns is almost constant when the temperature is lower than 80 °C (*R*/*R*_0_ value at 80 °C is only around 1.3), indicating their acceptable thermal stability. In bending, these patterns also have a similar relative resistance if bending radius is larger than 6 mm (Fig. 4d), while the rewritten pattern has a slightly higher resistance change if the radius is smaller than 5 mm. This may be because the rewritten pattern at a similar *N*_rew_ owns a more serious surface cracks (inset in Fig. 4d), which are easier to cause crack prorogation during bending. Reducing the *N*_rew_ in rewriting can decrease the cracking tendency, thereby achieving a better bending performance of the pattern. For example, the *R*/*R*_0_ value of the 8 *N*_rew_ pattern is about 2.9 (3.07 vs. 5.93) lower than of 10 *N*_w_ pattern after 2000 bending cycles at 3 mm radius. Notably, the relative resistance of all these patterns is nearly constant if the bending radius is larger than 6 mm. In situ monitoring during the bending cycles indicates the resistance of patterns increases in bending state and rapidly back to the initial value in recovery (Fig. 4e), with a change period agrees with the bending frequency. The rapid response and high durability provide the pattern with a potential of servicing as the functional components in electronics, such as strain sensors. It also indicates the presented sustainable technique is suitable for the development of complex micro-electro-mechanical structures, which can be achieved by a more precise laser controlling.

**Fig. 5:**
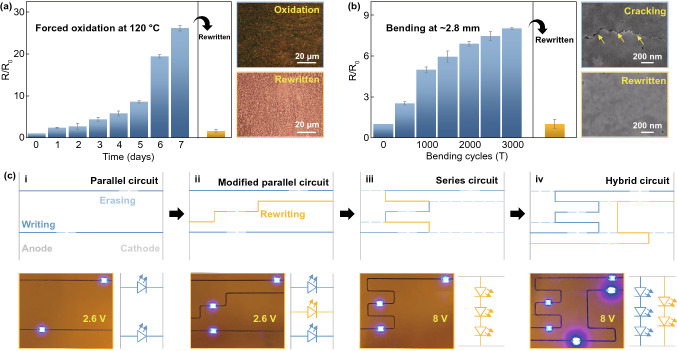
Demonstration of erasable laser writing for repairing and reprogramming circuits. Relative resistance changes of the patterns during **a** enforced oxidation process, **b** bending process, and after rewriting. Insets are the surface morphologies of the pattern, respectively, corresponding to the state after oxidation, bending, and rewriting. **c** Schematics of pattern changes and their corresponding actual digital images for the reprogrammed circuits. The subsequent circuits are gradual modifications from the first case but on different PI substrates. LEDs are introduced after finishing these circuits to confirm they are conductive

This erasing–rewriting process can remove the damaged patterns and reconstruct the conductive patterns. Figure 5a plots the relative resistance changes in an as-written pattern during the enforced oxidation at 120 °C. The relative resistance of the pattern increases as the storage time increases, which reaches around 26 after 7 days. Correspondingly, the surface of the pattern exhibits an oxidized dark green color (inset in Fig. 5a). XPS analysis of the oxidized pattern indicates its surface mainly consists of Cu_2_O (Fig. S16), thereby it still can be converted into the Cu^2+^ species into solution, i.e., being erased. Similarly, the pattern resistance increases as the bending cycles grow with a bending radius of 2.8 mm (Fig. 5b). The relative resistance change of the pattern after 3000 bending cycles is around eight because the cracking deteriorates its conductivity. After covering with the precursor, these patterns can be erased by irradiating a defocused laser, and then consequently impose the laser rewriting process to recovery the Cu pattern as fresh. The resistance of the rewritten patterns is close to that of the as-written pattern, confirming the suitability of this technology in circuit restoring.

This novel all-laser processing technology also provides a facile route for circuit modification in the manufacturing process. As shown in Fig. 5c-i, two parallel patterns are first written on the substrate to connect the external anode and cathode. Then, the regions where being required to place the electronic components can be removed by selective laser erasing process. A typical parallel circuit is obtained after placing two light-emitting diodes (LEDs) on the erased region, which can work at a bias voltage of 2.6 V. As shown in Fig. 5c-ii, the writing process can add an extra parallel in the circuit. When considering of changing such a parallel circuit into a series one as a proof-of-concept, the unnecessary parts of the circuit can be removed through the laser erasing process. Together with the rewriting process, a series circuit is fabricated successfully as shown in Fig. 5c-iii. Lighting up these series connected LEDs requires the bias voltage to be increased to 8 V. Owing to the fully removed original circuit, the erased region also can be rewritten with a new pattern to modify it into a complex one (Fig. 5c-iv). Here, the voltage distribution of a single LED on the two branches is not equal, resulting in a difference in their brightness.

## Conclusions

In summary, we have developed an erasable laser writing technology based on an acidic ionic Cu salt precursor that can reversibly manufacture the highly conductive Cu pattern on the target substrate. During the writing process, irradiating the focused laser induces a photothermal reaction at the region close to the substrate, which can decompose the reducing agent to manufacture the conductive Cu pattern. Irradiating a defocused laser can cause a thermally accelerated electrochemical corrosion of the Cu structure in the acidic precursor, thereby dissolving the as-written or invalid pattern to achieve the laser erasing process. The laser rewriting process can then be further employed to manufacture the conductive Cu pattern at these erased regions because of their near-initial state. The comparable resistivity, adhesion, thermal stability, and bending performance between the as-written and rewritten patterns verify the excellent reversibility of the proposed technology. Not only can this technology be used to manufacture and restore conductive patterns, but the proof-of-concept reprogrammable circuit also confirms its availability for dynamic adjustment of the circuit.

## Supplementary Information

Below is the link to the electronic supplementary material.

Supplementary file1 (DOC 10442 kb)
