# Supplementary material for: Laser Erasing and Rewriting of Flexible Copper Circuits
Source: Nanomicro Lett. 2021 Aug 31;13:184. doi: 10.1007/s40820-021-00714-3 (PMC8408303; doi:10.1007/s40820-021-00714-3)
Supplement: Supplementary file 1 — Supplementary file1 (DOC 10442 kb) [file 40820_2021_714_MOESM1_ESM.docx]

# Supporting information

*for*

**Laser Erasing and Rewriting of Flexible Copper Circuits**

Xingwen Zhou^1^, Wei Guo^1^, Peng Peng^2*^

1 School of Mechanical Engineering and Automation, Beihang University, Beijing 100191, China

2 Centre for Advanced Materials Joining, Department of Mechanical and Mechatronics Engineering, University of Waterloo, Waterloo, ON N2L 3G1, Canada

* Corresponding author: [peng.peng@uwaterloo.ca](mailto:peng.peng@uwaterloo.ca)

## Section 1. Typical liquid precursor film and erasing of the pattern on the glass substrate

**Figure S1** shows the digital image of a typical liquid film formed on a glass substrate. As seen, the liquid film has a convex top surface with a curved edge because of the surface tension. The laser processing should be performed at the central region where the liquid-thick (around 0.5 mm) is relatively uniform. Furthermore, because the laser-induced thermocapillary flow will results in local depression during laser scanning [1], the next laser scanning cycle should be performed after the liquid film has recovered naturally.


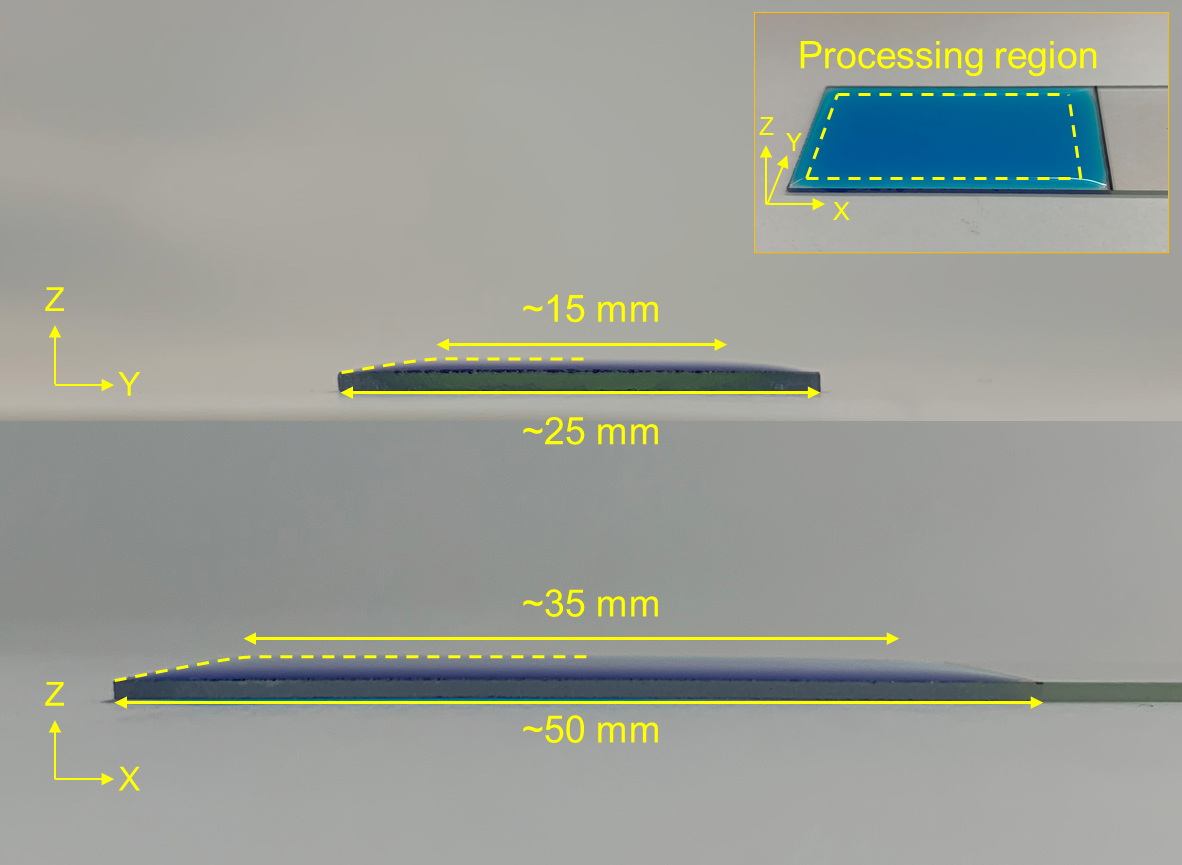


**Fig. S1** Digital image of the liquid film formed on the glass substrate, showing a relative plat region at the center (around 35 ×15 mm).

**Figure S2** shows the typical OM images of the laser written Cu patterns (5 *N*_w_) and after erasing (60 *N*_e_). No pattern on the erased region can be observed indicates the applicability of laser erasing to glass substrate. Notably, PI is highly recommended as the substrate for the proposed laser processing because of its flexibility and better adhesion to the pattern.


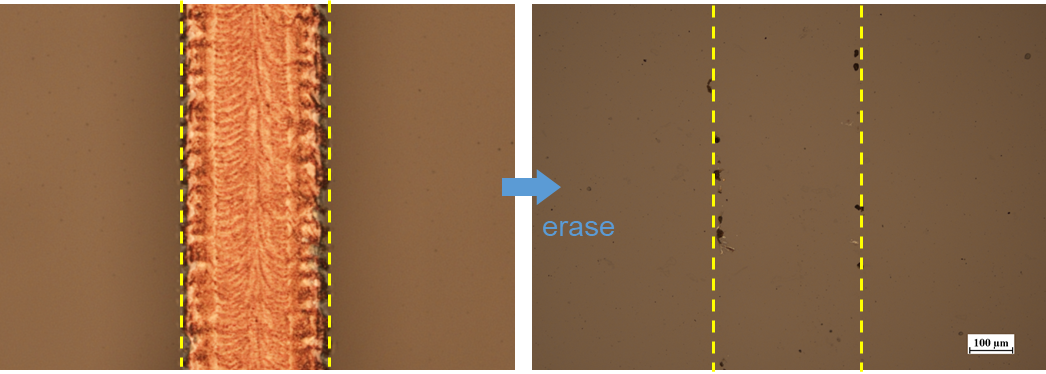


**Fig. S2** Optical images of (a) the laser written pattern on the glass substrate and (b) the region after erasing. Laser power for writing ≈12.6 W, Laser power for erasing ≈2.7 W, *N*_w_ = 5, *N*_e_ = 60.

## Section 2. Changes of the precursor during the laser irradiating processes

Direct irradiation of the precursor was performed by irradiating a laser with a spot diameter of about 3 mm on the side of the glass vial (see inset photo of **Fig. S3a**). The precursor has been diluted to 1/200 using EG to ensure sufficient reducing agent content in the solution. During this process, the peak temperature of the solution slowly increases and constant at around 140℃ within 8 min, and then rapidly increases to around 400℃ whin 10 s (see **Fig. S3a**). This steep temperature can be ascribed to the increased energy absorption due to the rapid formation of Cu layer. Notably, only the temperature around laser irradiation position can reach such a high temperature, while the temperature of bulk solution is still constant at around 120℃ (see **Fig. S3b**).


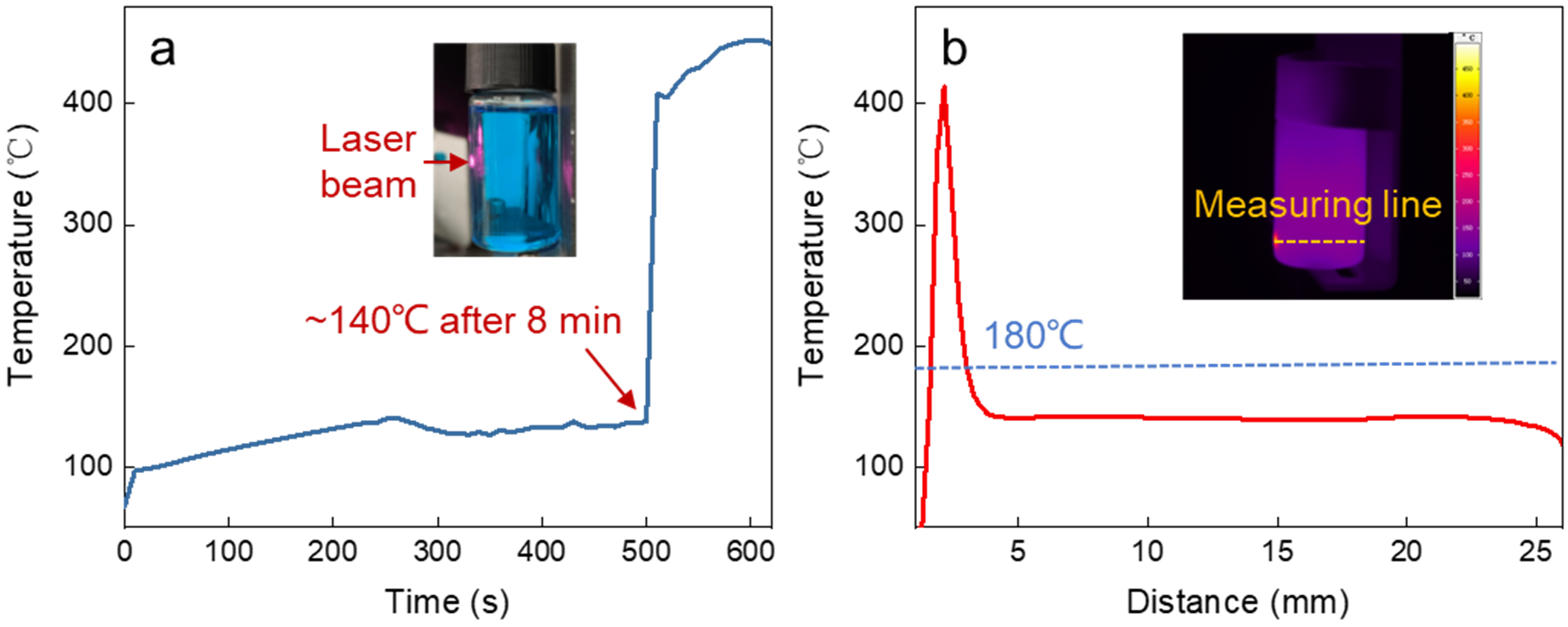


**Fig. S3** (a) Peak temperature change of the solution during laser irradiating. Inset is the digital photo for a typical experimental setup. (b) Temperature distribution along the laser passageway. Inset is the corresponding infrared camera image.

After laser irradiation, the products in solution are filtrated and dried at room temperature for XRD analysis. The peak at around 36.5°, 42.2°, 61.5°, and 73.7° are corresponding well to the (111), (200), (220), and (311) planes of cubic structured Cu_2_O (PDF #77-0199), indicating the products mainly consist of Cu_2_O (see **Fig. S4**). Notably, there are **NO** peaks attributed to cubic structured Cu (PDF #65-9743; typical peaks at 43.4°, 50.6°, and 74.3°) are identified, indicating the formation of Cu structure is negligible in the solution due to the insufficient temperature.


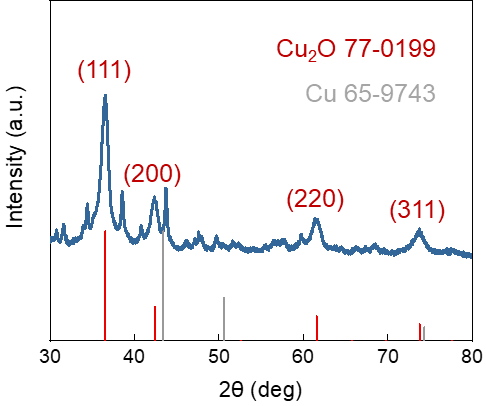


**Fig. S4** XRD result of the products in precursor after laser irradiating. Uncalibrated peaks may be related to other complexes.

Direct irradiation of the precursor (without dilution) was also performed in the absence of any solid medium along the laser passageway to reveal the substrate influence. As shown in **Fig. S5a**, the temperature of precursor filled in 45 ×12 mm cuvette immediately begins to rise when irradiating a focused laser on its top surface (~650 μm spot diameter and 4.7 W laser, agrees with writing on the PI substrate). The temperature distribution along the laser passageway indicates the significant temperature rise depth is ~1.7 mm after ~0.016 s (a single minimum sampling interval, see **Fig. S5b**). This indicates temperature penetration can reach a depth larger than the thickness of our liquid precursor (~0.5 mm, see **Fig. S1** above) within a short time. Both the peak temperature and temperature penetration increase as the irradiating time increases, whereof the depth of temperature penetration can reach around 31 mm after ~167 s (see **Fig. S5c**). However, the temperature rise rate of solution is extremely low to reach the decomposition temperature (~115℃ after ~167 s) of the reducing agent in a short time. This may be because the heat transfer in solid is mainly contributed by the thermal conduction, while it can be dominated by the additional thermal convection in liquid. The temperature rise rate when processing on the substrate can be significantly larger than that in bulk liquid because of the rapid local accumulation of heat around the substrate and limited precursor volume.


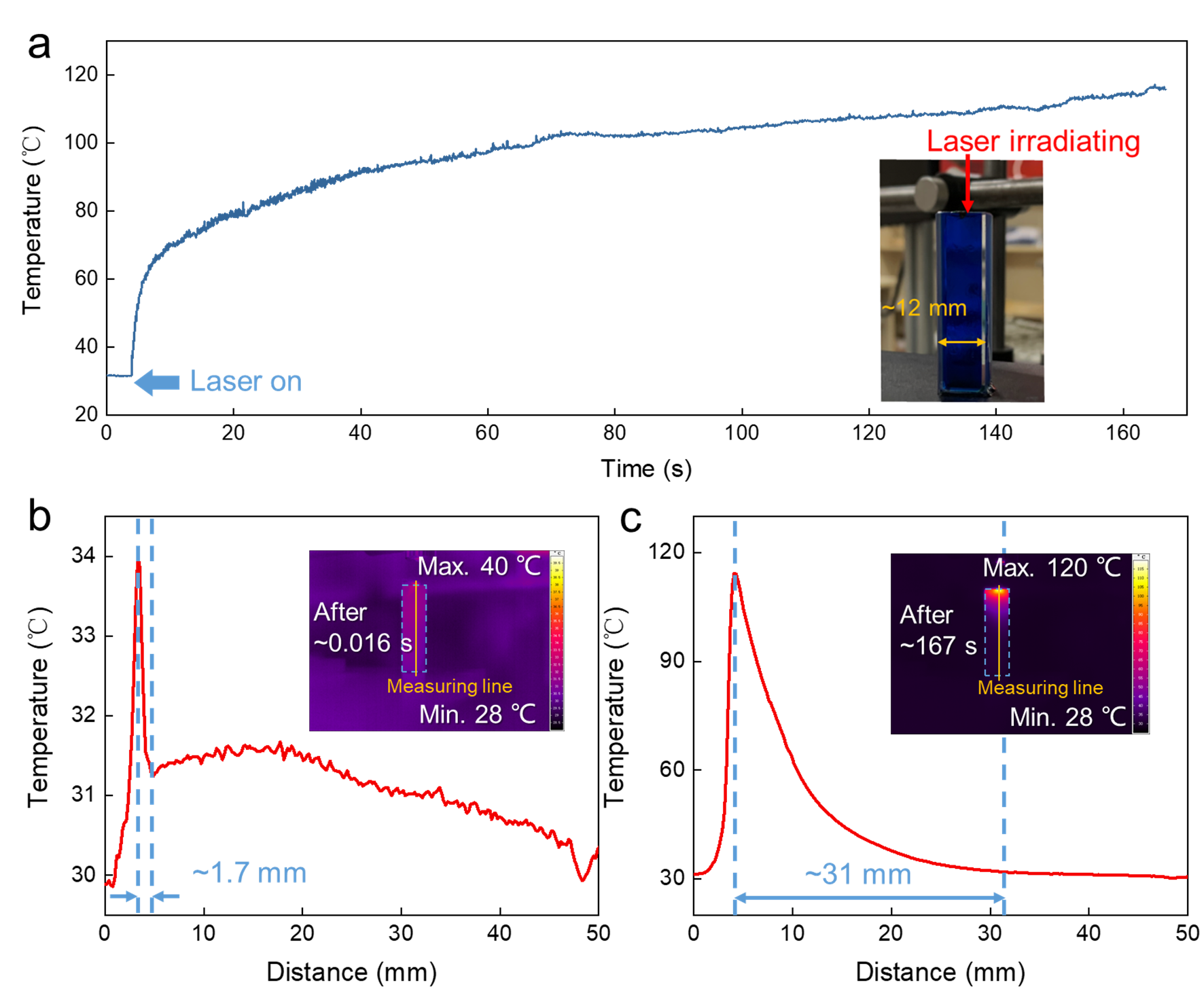


**Fig. S5** (a) Peak temperature change of the solution during laser irradiating. Temperature distribution along the laser passageway respectively at (b) ~0.016 s and (c) ~167 s. The precursor composition is agreed with that used for processing on the substrate.

## Section 3. Parametric optimization of laser writing on the PI substrate

In the writing process, voids and cracks are two typical defects observed in the pattern. Voids generally have smooth edges, while cracks own jagged edges and are continuously distributed on the pattern surface. **Fig. S6** shows the SEM images of the Cu patterns obtained at different *N*_w_. Some voids can be observed in the pattern obtained after 5 *N*_w_ is porous because of the insufficient amount of reduced Cu nanoparticles [2]. These voids are almost invisible after 10 *N*_w_, while a few cracks emerge on the surface of pattern because of the accumulation of thermal stress. The number, length, and displacement of the cracks increase as the *N*_w_ increases to 15.


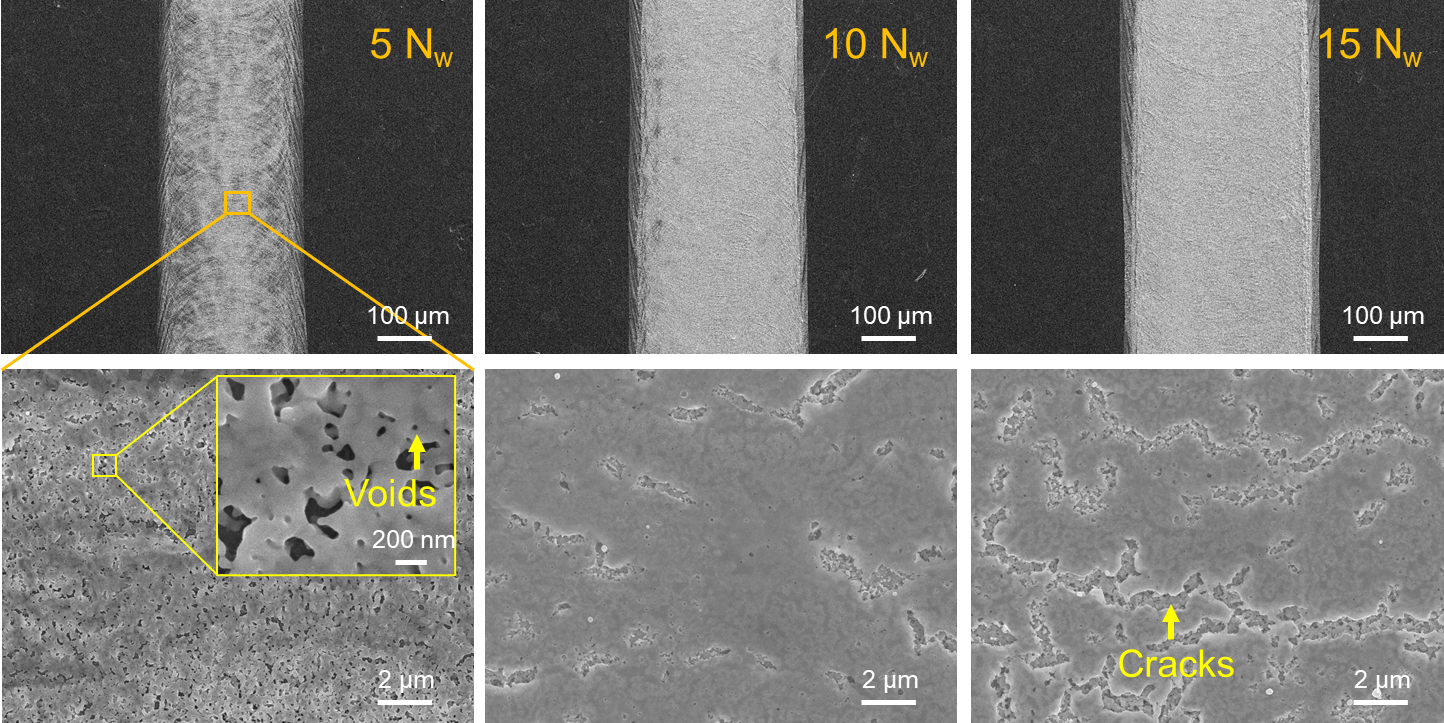


**Fig. S6** SEM images showing the microstructures of Cu patterns as a function of the *N*_w_.

**Figure S7a** plots the thickness and width of the patterns as a function of *N*_w_, showing the volume of patterns increases as the *N*_w_ increases. This is because the previously formed porous Cu and increase in absorbance of local precursor can absorb more laser energy to cause a higher local temperature rise [2]. However, the thickness of pattern is almost constant at around 0.9 μm after 15 *N*_w_, indicating it reaches a saturation value. This can be explained by the saturated heat capacity around the substrate [3] and/or reaching the thickness of the liquid actually involved in the reaction, which needs further confirmation. As shown in **Fig. S7b**, the resistivity of pattern is positively related to its densification. The resistivity of pattern drops to a similar order of magnitude as bulk Cu after 5 *N*_w_ because of the densification of structure. The magnitude of the resistivity is constant as the *N*_w_ further increases (it slightly increases after 15 *N*_w_ due to the increased surface cracks).


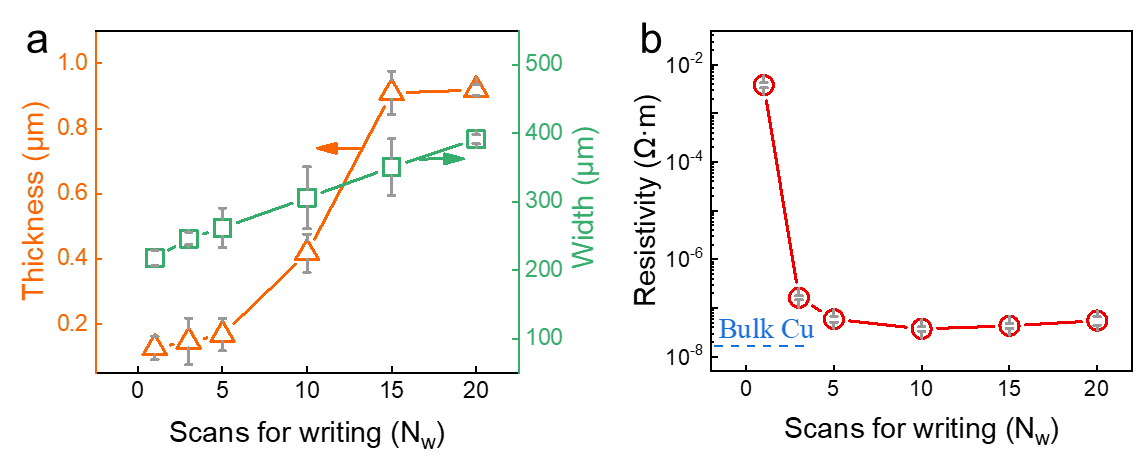


**Fig. S7** (a) Widths and thicknesses of the patterns (measured according to the cross-sectional SEM images) as a function of the scans for writing. (b) Resistivities of the patterns after various scans for writing. The resistivity of the pattern obtained at 10 *N*_w_ is around 3.7 Ω·m.

**Figure S8** plots the atomic concentration of the pattern obtained at 10 *N*_w_ as a function of sputter depth. The atomic concentration of Cu is around 17 % on the surface of pattern, while it sharply increases to 90% after sputtering about 5 nm, and constants at around 95% as the depth further increases. These results indicate the pattern mainly consists of pure Cu.


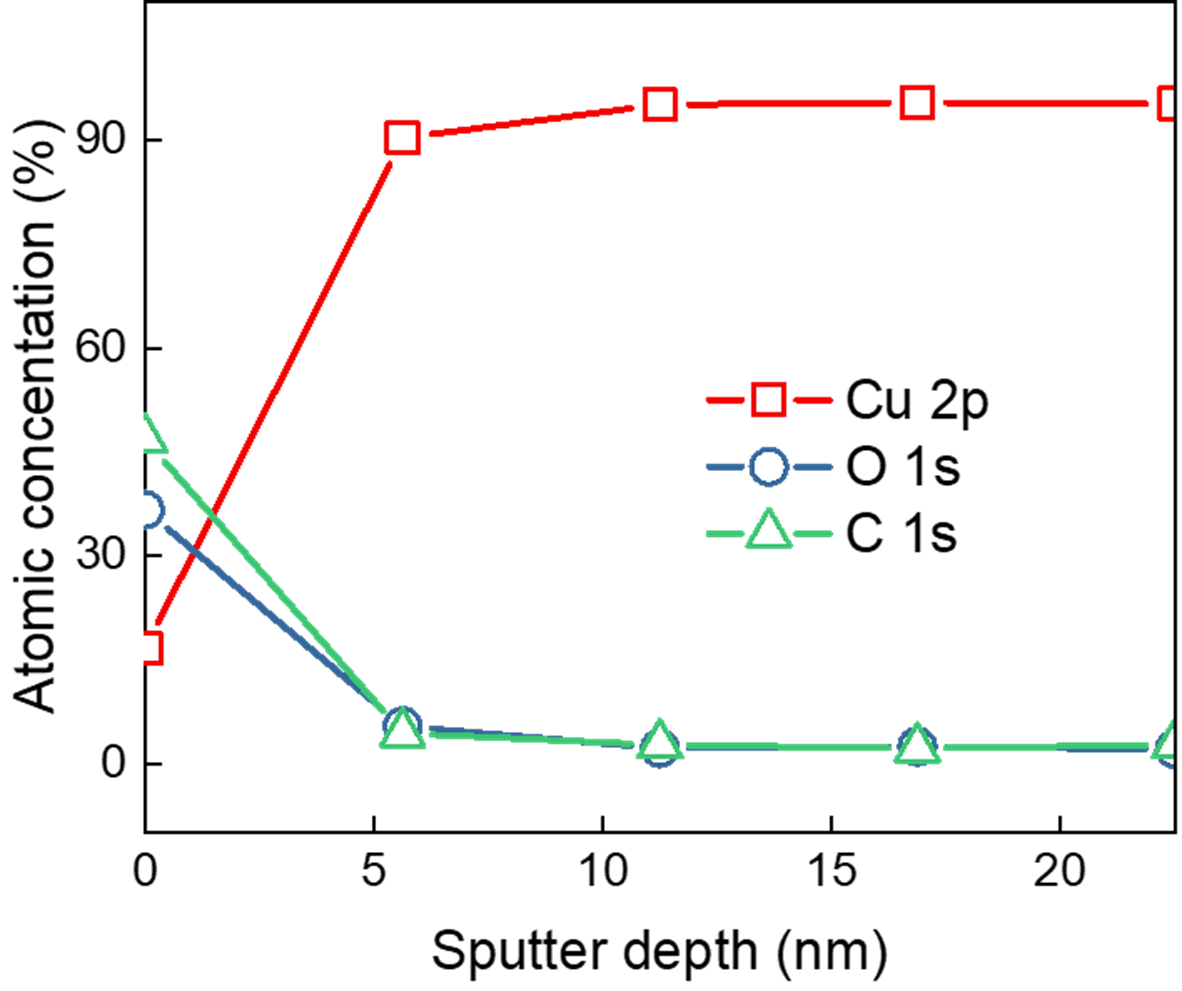


**Fig. S8** Atomic concentration of the pattern obtained at 10 *N*_w_ as a function of sputter depth

## Section 4. Parametric study of the laser erasing process on PI substrate

**Figure S9a** lists the OM images of the Cu patterns as a function of the *N*_e_. The erasing is an uneven process in which the region at irradiating center is dissolved first due to the Gaussian distribution of laser beam. The color of the pattern in the erased region turns to dark black after 20 *N*_e_, suggesting the transform of Cu to Cu^1+^/Cu^2+^ species on the pattern surface [4]. As the *N*_e_ increases to 80, the pattern can be fully erased. **Figure S9b** displays the OM images of the Cu patterns after erased 80 *N*_w_ at different defocus distances. The erased width of the pattern can be reduced to around 600 μm as the defocus distance decreases to 8 mm. However, the structure still presents at the irradiated center if further decreasing the defocus distance to 7 mm (as the arrow marked in **Fig. S9b**). **Figure S10** lists the typical SEM images of the patterns as a function of the *N*_e_ (Center region in **Fig. S9a**). The Cu pattern obviously decreases as the *N*_e_ increases. These residual Cu-rich structures are surround by the organics. Only the rough PI substrate can be observed after 60 *N*_e_.


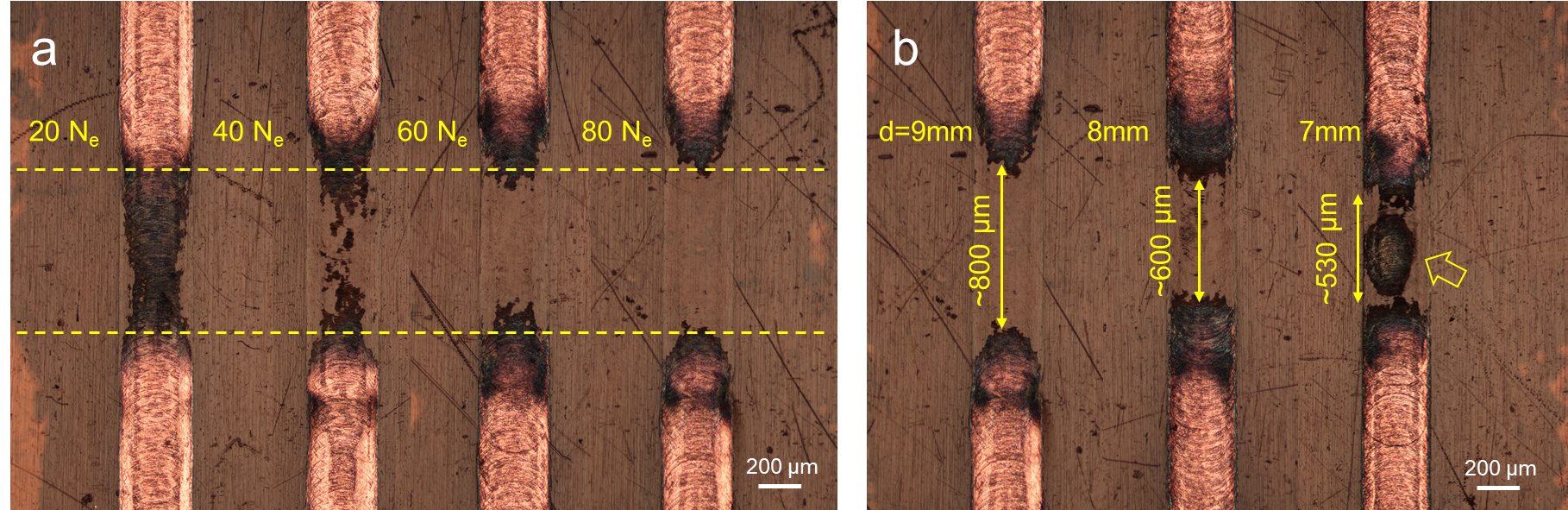


**Fig. S9** OM images showing the microstructures of Cu patterns (a) as a function of the *N*_e_ and (b) erased after 80 *N*_e_ with different defocus distances.


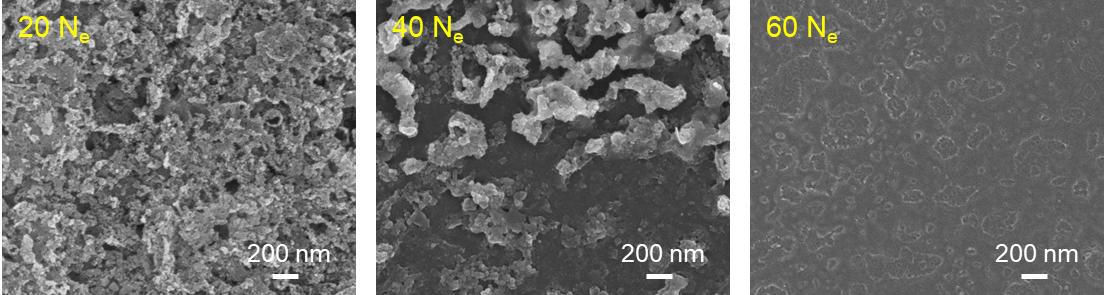


**Fig. S10** SEM images showing the change of Cu patterns as a function of the *N*_e_.

SEM image in **Fig. S11a** indicates the residual pattern obtained at the 7 mm defocus distance is dense sintered structure. A peak around 917.7 eV is identified in the Cu LMM spectrum, which agrees well with the position of CuO (see **Fig. S11b**). In the O 1s spectrum, the CuO peak at 529.7 eV is corresponding identified, confirming the formation of CuO (see **Fig. S11c**). The peak at around 531.8 eV in the O 1s spectrum is related to the C=O bond or chemical absorbed oxygen on the pattern surface. The peak at around 533 eV in the O 1s spectrum is assigned to the C-O- bond.


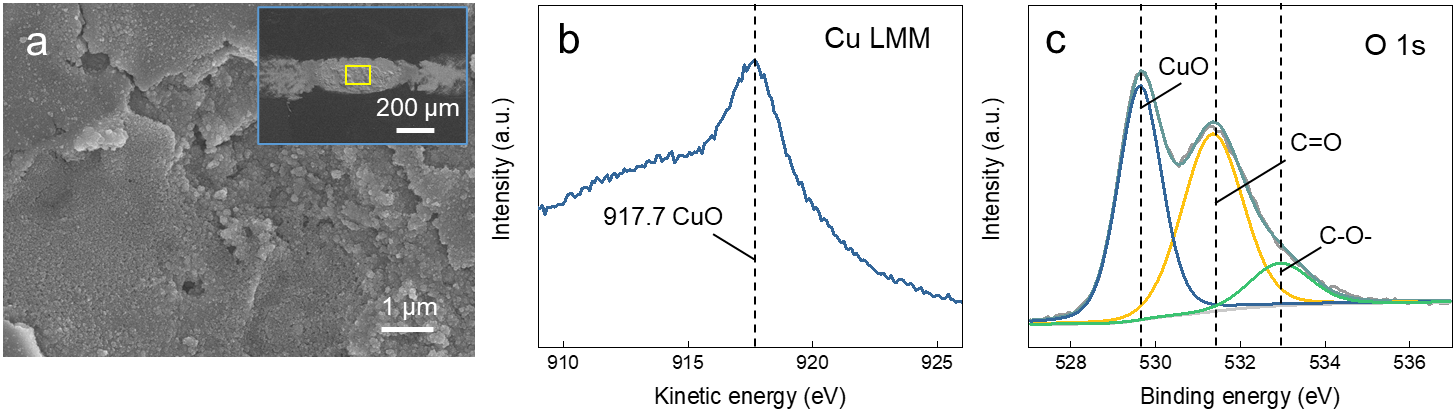


**Fig. S11** (a) SEM image, (b) Cu LMM spectrum, and (c) O 1 s spectrum of the pattern after laser erasing with a defocus distances of 7 mm. *N*_e_ = 80.

The dark-color region at the edge of erased region is induced by the multiple thermal cycles at the edge of laser irradiation (i.e. the heat affected zone of laser processing). As shown in **Fig. S12**, the residual pattern at these regions can be converted into the fresh Cu by the rewriting process because of their large surface area [5], thereby showing no negative effect on the conductivity of rewritten pattern.


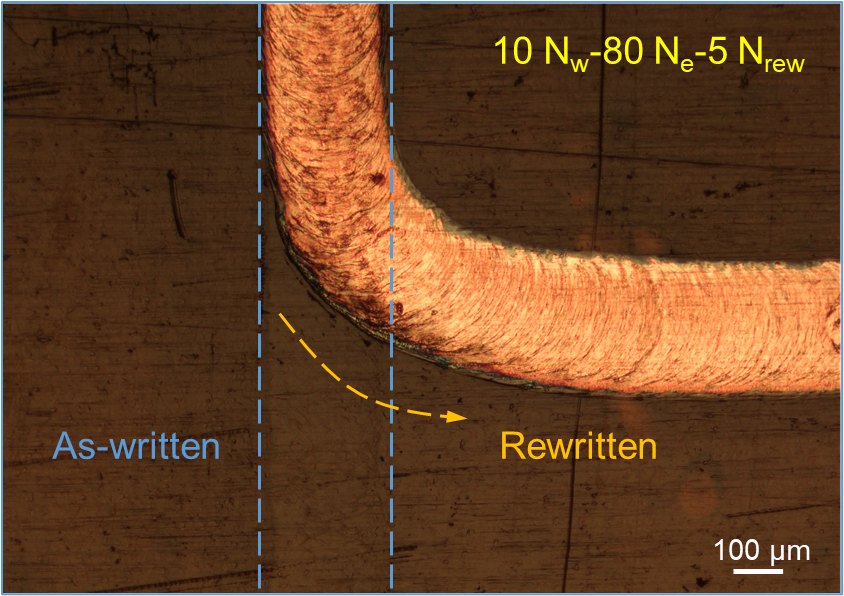


**Fig. S12** OM image showing the microstructures of the 10 *N*_w_ pattern after rewriting.

## Section 5. Comparison between the erased and fresh regions

**Figure S13** compares the XPS spectrum of the fresh and erased regions on the PI substrate. The compositions have been calculated based on the relative areas of the identified peaks. The atomic concentration of the fresh region is 66.25 C 1s-6.63 N 1s-26.96 O 1s-**0.15 Cu 2p3** (%), while it is 68.76 C 1s-8.92 N 1s-21.56 O 1s-0.76 Cu 2p3 (%) in the erased region. **Figure S14** compares the micro-morphologies of the fresh and erased regions on the PI substrate, showing the erased region owns a higher roughness than the fresh region.


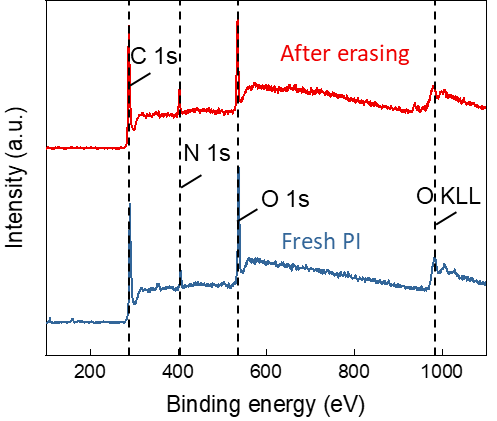


**Fig. S13** XPS spectrum of the erased and fresh regions on the PI substrate.


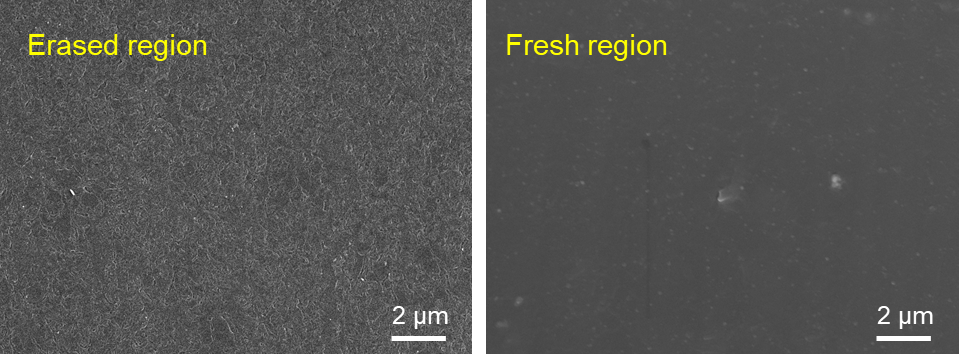


**Fig. S14** SEM images of the erased and fresh regions on the PI substrate.

## Section 6. Selectively thermal erasing process

**Figure S15** shows the typical OM images of the laser written Cu patterns and after selective erasing with the assistance of 3M tape. The tape is first covered on the sample to protect the desired region of the pattern. Then, the liquid precursor is covered on the sample and heating at 90℃ for about 20 min. After removing the precursor and tape, the covered pattern can remain on the substrate. Covering the patterns with 3M tape during laser erasing can also improve the flatness of their edges.


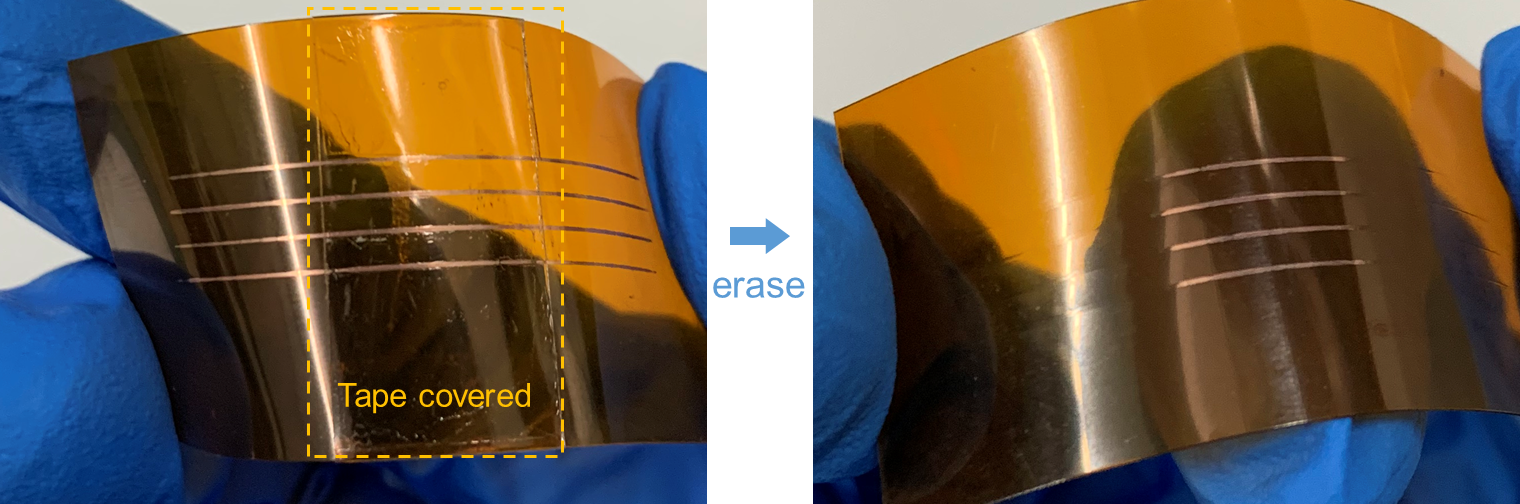


**Fig. S15** Selectively thermal erasing of the undesired region of the pattern with the assistance of 3 M tape.

## Section 7. Surface composition of the pattern after oxidation

**Figure S16** illustrates the XPS spectra of the pattern after enforced oxidation at 120℃. A peak around 916 eV is identified in the Cu LMM spectrum, which is close to the position of Cu_2_O (916.8 eV). In the O 1s spectrum, the Cu_2_O peak at 530.4 eV is corresponding identified, confirming the pattern surface consists of mainly Cu_2_O.


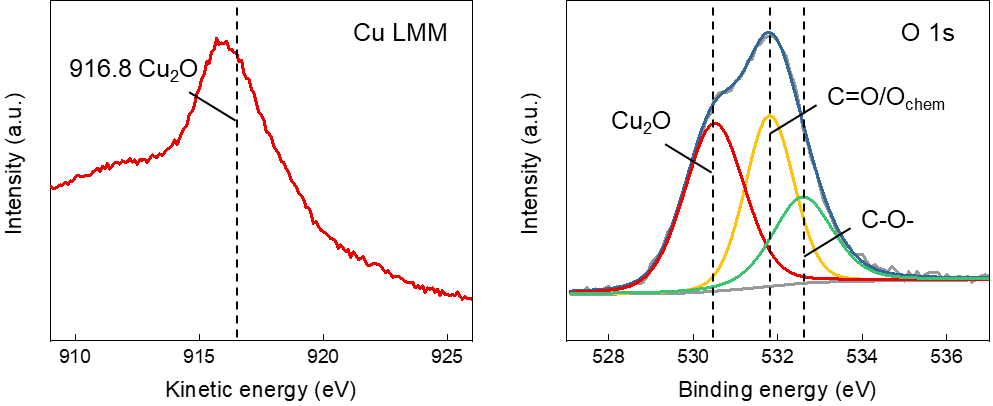


**Fig. S16** Cu LMM and O 1s spectra of the pattern after enforced oxidation at 120℃.

## References

1. H. M. J. M. Wedershoven, C. W. J. Berendsen, J. C. H. Zeegers, A. A. Darhuber. Infrared-laser-induced thermocapillary deformation and destabilization of thin liquid films on moving substrates. Physical Review Applied. **3**(2), 024005 (2015). <https://doi.org/10.1103/PhysRevApplied.3.024005>

2. X. Zhou, W. Guo, Y. Zhu, P. Peng. The laser writing of highly conductive and anti-oxidative copper structures in liquid. Nanoscale. **12**(2), 563-571 (2020). <https://doi.org/10.1039/c9nr07248a>

3. K. Kwon, J. Shim, J. O. Lee, K. Choi, K. Yu. Localized laser-based photohydrothermal synthesis of functionalized metal-oxides. Advanced Functional Materials. **25**(15), 2222-2229 (2015). <https://doi.org/10.1002/adfm.201404215>

4. K. F. Khaled, M. A. Amin. Dry and wet lab studies for some benzotriazole derivatives as possible corrosion inhibitors for copper in 1.0m hno3. Corrosion Science. **51**(9), 2098-2106 (2009). <https://doi.org/10.1016/j.corsci.2009.05.038>

5. S. Han, S. Hong, J. Yeo, D. Kim, B. Kang, M. Y. Yang, S. H. Ko. Nanorecycling: Monolithic integration of copper and copper oxide nanowire network electrode through selective reversible photothermochemical reduction. Advanced Materials. **27**(41), 6397-6403 (2015). <https://doi.org/10.1002/adma.201503244>
